# Dr. Atul Thakre

**Published:** 2009

**Authors:** Gopal Badlani

**Affiliations:** E-mail: gbadlani@wfubmc.edu

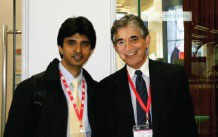



Death defies logic when a young, bright, good soul is taken away in his prime, leaving behind grieving family, friends but more importantly scores of patients who would have benefitted from his skillful hands.

Atul willed his way into the world of pediatric urology, more internationally than in India. I first met Atul in one of the urology camps in India. His incisive questions and precise skills in the OR were easily apparent. In my quest to have a trained pediatric urologist in India to care for scores of patients being seen with complex urological issues, I encouraged him to seek a career in Pediatric Urology. Unlike many others who “yes” me, he took the time to see Ped urology first hand by spending three months at LIJ. His enthusiasm was apparent to all he met in the world of Ped urology through LIJ and various meetings. Neeta his dear wife was as responsible for encouraging him to pursue his passion. With help from Craig Peters & others he secured a fellowship at the Prince of Wales Hospital in Hong Kong. C. K Youn was his teacher, and Atul was on his way.

Despite an offer to stay in Hong Kong, I was grateful that he kept his promise to me by coming back to India as the first fellowship trained pediatric urologist and helping the children with his talent.

While he was climbing the academic ladder, being invited to write chapters, review papers, speak at international meetings, to me he remained Atul who would not say no to a poor child I would refer to him for care. Very few in the world remain true to their word, he was one of them.

My emptiness pales in comparison to that of his wife Dr. Nita Thakre and his children Udit and Anuj.

